# Expression of IFITM1 as a prognostic biomarker in resected gastric and esophageal adenocarcinoma

**DOI:** 10.1186/s40364-016-0064-5

**Published:** 2016-05-14

**Authors:** David Borg, Charlotta Hedner, Alexander Gaber, Björn Nodin, Richard Fristedt, Karin Jirström, Jakob Eberhard, Anders Johnsson

**Affiliations:** Department of Clinical Sciences Lund, Division of Oncology and Pathology, Lund University, Skåne University Hospital, 221 85 Lund, Sweden

**Keywords:** Esophageal neoplasms, Stomach neoplasms, Adenocarcinoma, Prognosis, *IFITM1*

## Abstract

**Background:**

There is an increasing amount of reports on IFITM1 (interferon-inducible transmembrane protein 1) in various malignancies. The aim of this study was to examine the expression of IFITM1 and its prognostic significance in gastroesophageal adenocarcinoma.

**Methods:**

Tissue samples were obtained from a consecutive cohort of 174 patients surgically treated between 2006 and 2010 for gastroesophageal (gastric, gastroesophageal junction and esophageal) adenocarcinoma, not subjected to neoadjuvant therapy. Expression of IFITM1 was examined using immunohistochemistry on tissue microarrays of primary tumors and paired samples of adjacent normal epithelium, intestinal metaplasia and lymph node metastases.

**Results:**

Expression of IFITM1 was significantly elevated in primary tumors and lymph node metastases compared to adjacent normal epithelium and intestinal metaplasia, regardless of tumor location. Overexpression of IFITM1 was associated with M0-disease (no distant metastases). In gastric cancer IFITM1 expression was significantly associated with improved TTR (time to recurrence) in Kaplan-Meier analysis and Cox regression, both in the unadjusted analysis (HR 0.33, 95 % CI 0.12-0.88) and in the adjusted analysis (HR 0.32, 95 % CI 0.12-0.87) but there was no significant impact on OS (overall survival). In esophageal adenocarcinoma expression of IFITM1 had no impact on TTR or OS in Kaplan-Meier-analyses, but in the adjusted Cox regression IFITM1 expression had a negative impact on both TTR (HR 3.05, 95 % CI 1.09-8.53) and OS (HR 2.71, 95 % CI 1.11-6.67).

**Conclusions:**

IFITM1 was overexpressed in gastroesophageal adenocarcinoma and associated with M0-disease. In gastric cancer IFITM1 expression had a positive impact on TTR but in esophageal cancer it seemed to have an adverse impact on survival.

The reason for the diverging prognostic impact of IFITM1 in esophageal and gastric cancer is unclear and warrants further studies.

**Electronic supplementary material:**

The online version of this article (doi:10.1186/s40364-016-0064-5) contains supplementary material, which is available to authorized users.

## Background

Gastroesophageal adenocarcinoma is the 5th most common cancer worldwide [1]. The incidence of esophageal and GE (gastroesophageal) junction adenocarcinoma has drastically increased in many Western countries for the last four decades [2, 3]. Suggested factors to explain this increase are gastroesophageal reflux disease, obesity and decreased prevalence of *Helicobacter pylori* infection [4, 5]. In contrast, the incidence of gastric adenocarcinoma has declined globally for several decades [6], possibly due to decreased prevalence of *Helicobacter pylori* infection and improved dietary conditions [7].

The prognosis of gastroesophageal adenocarcinoma is generally poor, at least in Western populations. For operable patients with resectable tumors recent studies have shown that the addition of neoadjuvant and/or adjuvant chemotherapy or chemoradiotherapy improves the 5-year survival rate with 10–15 % [8–11]. To further improve the overall survival in gastroesophageal adenocarcinoma, a deepened understanding of the tumor biology is required. Moreover, identification of prognostic and response predictive biomarkers is warranted to optimize and personalize the treatment strategies.

IFITM1 (interferon-inducible transmembrane protein 1), also known as 9–27, Leu-13 or CD225, is a cell surface 17-kDa membrane protein that is encoded on the short arm of chromosome 11. It is mainly known as an inhibitor of viral entry and replication [12], but it has also been associated with angiogenesis [13], inflammatory bowel disease [14] and osteogenesis [15].

There are now emerging data on IFITM1 and its role in malignancy. An upregulation of IFITM1 in different types of cancer and promotion of tumorigenesis by enhancing tumor cell migration, invasion and proliferation has been reported in several studies [16–23] but the opposite has also been shown [24–26]. Overexpression of IFITM1 has been reported to correlate with improved survival in glioma and chronic myeloid leukemia [17, 27] but in a South Korean study on gastric cancer, there was a trend towards worse survival in patients with high expression of IFITM1 [23]. Apart from the latter study, the knowledge on IFITM1 in gastroesophageal cancer survival is very limited, especially in Western populations. Therefore, the current study was designed to explore the expression and prognostic significance of IFITM1 in adenocarcinoma of the esophagus, GE junction and stomach in a consecutive cohort of patients from southern Sweden, that were treated 2006–2010, prior to the wide implementation of (neo-)adjuvant oncological treatment.

## Methods

### Study design and participants

The study comprises a consecutive cohort of 174 patients with chemo-/radiotherapy-naive gastroesophageal (gastric, GE junction and esophageal) adenocarcinoma subjected to surgical resection at the University Hospitals of Lund and Malmö between January 1, 2006 and December 31, 2010. This patient cohort has been used in several previous reports on other biomarkers [28–32]. Data on survival and recurrence were updated until December 31 2014. Tumor location was based on endoscopy findings. Classification of tumor stage was done according to UICC/AJCC TNM edition 7. Residual tumor status was classified as: R0 = no residual tumor, R1 = microscopic residual tumor, R2 = macroscopic residual tumor. The vast majority of the patients were operated on with a curative intent but three patients with metastatic disease were resected to palliate symptoms from the primary tumor. In 16 patients, M1-disease (distant metastases) was revealed either during surgery or in the resected specimens. All patients had surgery up-front, without neoadjuvant oncological therapy and a minority (7.5 %) of the patients received adjuvant treatment (chemo-/radiotherapy). Clinical data, recurrence status and vital status were obtained retrospectively from medical records. Clinicopathological data and follow-up data are described in Table 1. The study was approved by the regional ethics committee at Lund University (ref nr 445/07).

**Table 1:** Patient and tumor characteristics

Factor	Entire cohort	Esophagus	GE junction	Stomach
	(*n* = 174) *n* (%)	(*n* = 60) *n* (%)	(*n* = 45) *n* (%)	(*n* = 69) *n* (%)
Age (years)				
Mean Median (Range)	70.2 70.0 (42.6-94.4)	67.9 66.0 (48.2-88.5)	69.9 68.7 (48.7-88.6)	72.4 73.9 (42.6-94.4)
Sex				
Women Men	40 (23.0) 134 (77.0)	6 (10.0) 54 (90.0)	12 (26.7) 33 (73.3)	22 (31.9) 47 (68.1)
T stage				
T1 T2 T3 T4 Unknown	19 (10.9) 32 (18.4) 93 (53.4) 27 (15.5) 3 (1.7)	9 (15.0) 10 (16.7) 34 (56.7) 6 (10.0) 1 (1.7)	3 (6.7) 4 (8.9) 33 (73.3) 4 (8.9) 1 (2.2)	7 (10.1) 18 (26.1) 26 (37.7) 17 (24.6) 1 (1.4)
N stage				
N0 N1 N2 N3	59 (33.9) 30 (17.2) 41 (23.6) 44 (25.3)	15 (25.0) 11 (18.3) 15 (25.0) 19 (31.7)	12 (26.7) 7 (15.6) 14 (31.1) 12 (26.7)	32 (46.4) 12 (17.4) 12 (17.4) 13 (18.8)
Number of examined nodes				
Mean Median Range Unknown	30.3 29.0 1–112 14	36.6 33.5 10–72 2	29.7 28.0 8–48 1	24.3 22.0 1–112 11
M stage				
M0 M1	152 (87.4) 22 (12.6)	52 (86.7) 8 (13.3)	40 (88.9) 5 (11.1)	60 (87.0) 9 (13.0)
R classification				
R0 R1 R2	121 (69.5) 43 (24.7) 10 (5.7)	38 (63.3) 21 (35.0) 1 (1.7)	30 (66.7) 13 (28.9) 2 (4.4)	53 (76.8) 9 (13.0) 7 (10.1)
Differentiation grade				
High Intermediate Low	8 (4.6) 53 (30.5) 113 (64.9)	4 (6.7) 26 (43.3) 30 (50.0)	1 (2.2) 13 (28.9) 31 (68.9)	3 (4.3) 14 (20.3) 52 (75.4)
Lauren classification				
Intestinal Mixed Diffuse	120 (69.0) 9 (5.2) 45 (25.9)	54 (90.0) 4 (6.7) 2 (3.3)	31 (68.9) 3 (6.7) 11 (24.4)	35 (50.7) 2 (2.9) 32 (46.4)
Intestinal metaplasia background				
No Yes	101 (58.0) 73 (42.0)	37 (61.7) 23 (38.3)	34 (75.6) 11 (24.4)	30 (43.5) 39 (56.5)
Adjuvant therapy				
No Chemoradiotherapy Chemotherapy Radiotherapy	161 (92.5) 11 (6.3) 1 (0.6) 1 (0.6)	55 (91.7) 3 (5.0) 1 (1.7) 1 (1.7)	42 (93.3) 3 (6.7)	64 (92.8) 5 (7.2)
Follow-up (years)				
Mean Median Range	3.25 2.28 0.01-8.95	3.36 2.47 0.26-8.95	3.06 2.17 0.01-8.89	3.28 2.09 0.01-8.85
Recurrence				
No Yes Unknown/Not applicable	62 (35.6) 78 (44.8) 34 (19.5)	20 (33.3) 29 (48.3) 11 (18.3)	15 (33.3) 22 (48.9) 8 (17.8)	27 (39.1) 27 (39.1) 15 (21.7)
Vital status				
Alive Dead	48 (27.6) 126 (72.4)	21 (35.0) 39 (65.0)	8 (17.8) 37 (82.2)	19 (27.5) 50 (72.5)

### Tissue microarrays

Using a semi-automated arraying device (TMArrayer™, Pathology Devices, Westminster, MD, USA) tissue microarrays (TMAs) were constructed. From all 174 primary tumors duplicate cores (1 mm) were obtained from areas with morphologically viable cancer in different blocks. In 81 cases lymph node metastases were sampled in duplicate cores. In addition 1–3 cores from intestinal metaplasia (gastric intestinal metaplasia or Barrett’s esophagus) were sampled in 73 cases. Single core samples from adjacent normal gastric mucosa (131 cases) and normal squamous epithelium of the esophagus (96 cases) were also retrieved. All samples were paired.

### Immunohistochemistry

For immunohistochemical analysis of IFITM1 expression, 4 μm TMA-sections were automatically pre-treated using the PT Link system and then stained in an Autostainer Plus (DAKO; Glostrup, Copenhagen, Denmark) with the rabbit polyclonal anti-IFITM1 antibody HPA004810 (Atlas Antibodies AB, Stockholm, Sweden) diluted 1:250. The specificity of the antibody has been validated [33]. Staining was assessed by two different observers (DB and AG) blinded to clinical and outcome data. Scoring discrepancies were discussed to reach consensus. IFITM1 staining was mainly detected in the cytoplasm, with an accentuation towards the membrane. The fraction of stained tumor cells was scored as: 0 (0–1 %), 1 (2–25 %), 2 (26–50 %), 3 (51–75 %) or 4 (>75 %). Staining intensity was scored as: 0 (negative), 1 (weak), 2 (moderate) or 3 (strong). By multiplying fraction and intensity a combined score (0–12) was constructed.

### Statistical analysis

The Mann–Whitney U test was applied to compare the distribution of IFITM1 expression in different tissues (Fig. 2) and also to describe the relationship between IFITM1 expression and clinicopathological factors (Table 2). Time to recurrence (TTR) was defined as time from date of surgery to date of biopsy or radiology proven recurrent disease. Overall survival (OS) was defined as time from date of surgery to date of death. TTR and OS were analysed for resected M0-patients with no macroscopic residual tumor (R0-1). To determine the optimal prognostic cut-off for IFITM1 expression in the primary tumors, ROC-curves were used. Differences in Kaplan-Meier survival curves were calculated by log-rank test (Fig. 3). Unadjusted and adjusted hazard ratios for survival were determined using Cox proportional-hazards regression (Table 3). The adjusted model for TTR included T-stage, N-stage and R-classification. For OS, the adjusted model included age, T-stage, N-stage, R-classification and differentiation grade. All tests were 2-sided and a *p*-value <0.05 was considered significant. IBM® SPSS® Statistics version 22.0.0.1 for Mac was used for all statistical analyses.

**Table 2:** Associations of IFITM1 expression in primary tumors with clinicopathological data

Factor	Entire cohort median (range)	*p*-value	Esophagus median (range)	*p*-value	GE junction median (range)	*p*-value	Stomach median (range)	*p*-value
Age								
≤ average >average	1.75 (0.00-10.50) 2.00 (0.00-12.00)	0.103	2.00 (0.00-10.50) 1.00 (0.00-7.50)	0.363	2.50 (0.00-9.80) 2.50 (0.00-12.00)	0.693	0.00 (0.00-9.00) 2.50 (0.00-12.00)	0.001
Sex								
Female Male	2.50 (0.00-11.00) 1.00 (0.00-12.00)	0.207	4.00 (1.00-6.50) 1.00 (0.00-10.50)	0.080	2.50 (0.00-6.50) 2.00 (0.00-12.00)	1.000	2.00 (0.00-11.00) 1.00 (0.00-12.00)	0.588
T-stage								
T1 T2 T3 T4	1.75 (0.00-10.00) 2.00 (0.00-12.00) 1.75 (0.00-12.00) 1.00 (0.00-12.00)	0.805	2.00 (0.00-8.00) 2.25 (0.00-7.50) 1.25 (0.00-9.80) 0.50 (0.00-7.00)	0.400	1.75 (0.00-3.50) 0.50 (0.00-4.00) 2.50 (0.00-12.00) 2.50 (2.00-8.00)	0.669	1.00 (0.00-10.00) 3.00 (0.00-12.00) 1.25 (0.00-11.00) 1.00 (0.00-12.00)	0.883
N-stage								
N0 N1 N2 N3	2.00 (0.00-12.00) 2.00 (0.00-12.00) 1.00 (0.00-12.00) 1.75 (0.00-10.50)	0.585	2.25 (0.00-8.00) 2.00 (0.00-6.50) 0.75 (0.00-9.80) 1.50 (0.00-10.50)	0.471	2.50 (0.00-12.00) 3.00 (0.00-11.00) 1.25 (0.00-8.00) 2.75 (0.00-9.80)	0.694	1.25 (0.00-12.00) 2.50 (0.00-12.00) 2.00 (0.00-12.00) 0.50 (0.00-6.00)	0.504
M-stage								
M0 M1	2.00 (0.00-12.00) 0.50 (0.00-6.50)	0.033	1.75 (0.00-10.50) 1.25 (0.00-5.50)	0.528	2.50 (0.00-12.00) 2.00 (0.00-6.50)	0.693	2.00 (0.00-12.00) 0.00 (0.00-2.00)	0.011
R-classification								
R0 R1 R2	2.00 (0.00-12.00) 1.00 (0.00-9.80) 0.00 (0.00-10.00)	0.055	2.00 (0.00-10.50) 1.00 (0.00-9.80) 0.00 (0.00-0.00	0.444	3.00 (0.00-12.00) 2.00 (0.00-7.00) 0.125 (0.00-0.30)	0.252	2.00 (0.00-12.00) 0.50 (0.00-4.50) 0.25 (0.00-10.00)	0.225
Differentiation grade								
High Intermediate Low	2.00 (0.00-3.00) 2.00 (0.00-12.00) 1.50 (0.00-12.00)	0.759	2.00 (1.00-2.00) 2.00 (0.00-10.50) 1.00 (0.00-8.00)	0.629	2.50 (2.50-2.50 1.50 (0.00-5.50) 2.50 (0.00-12.00)	0.586	0.50 (0.00-3.00) 3.00 (0.00-12.00) 1.00 (0.00-12.00)	0.324
Lauren classification								
Intestinal Mixed Diffuse	2.00 (0.00-12.00) 1.00 (0.00-6.50) 0.50 (0.00-12.00)	0.150	1.75 (0.00-10.50) 1.875 (0.00-6.50) 0.00 (0.00-0.00)	0.191	2.00 (0.00-12.00) 3.00 (0.00-3.00) 3.50 (0.00-12.00)	0.565	3.00 (0.00-12.00) 0.25 (0.00-0.50) 0.38 (0.00-10.00)	0.008
Intestinal metaplasia background								
No Yes	1.00 (0.00-12.00) 2.25 (0.00-12.00)	0.083	1.00 (0.00-10.50) 2.00 (0.00-7.00)	0.446	2.00 (0.00-12.00) 4.25 (0.00-12.00)	0.090	0.75 (0.00-12.00) 1.75 (0.00-12.00)	0.271
Location								
Esophagus GE junction Stomach	1.50 (0.00-10.50) 2.50 (0.00-12.00) 1.00 (0.00-12.00)	0.829

**Table 3:** Hazard ratios for recurrence and death M0 R0-1

	Time to recurrence				
		Unadjusted		Adjusted^a^	
	n (events)	HR (95 % CI)	*p*-value	HR (95 % CI)	*p*-value
Esophagus					
IFITM1					
Low High	23 (13) 16 (9)	1.00 1.09 (0.47-2.56)	0.836	1.00 3.05 (1.09-8.53)	0.034
GE junction					
IFITM1					
Low High	16 (10) 17 (10)	1.00 1.09 (0.45-2.62)	0.852	1.00 1.50 (0.59-3.82)	0.400
Stomach					
IFITM1					
Low High	30 (19) 20 (5)	1.00 0.33 (0.12-0.88)	0.026	1.00 0.32 (0.12-0.87)	0.026
	Overall survival				
		Unadjusted		Adjusted^b^	
	n (events)	HR (95 % CI)	*p*-value	HR (95 % CI)	*p*-value
Esophagus					
IFITM1					
Low High	31 (20) 18 (11)	1.00 0.99 (0.47-2.07)	0.976	1.00 2.71 (1.11-6.67)	0.029
GE junction					
IFITM1					
Low High	19 (15) 19 (16)	1.00 1.00 (0.49-2.03)	0.995	1.00 0.97 (0.44-2.15)	0.937
Stomach					
IFITM1					
Low High	32 (22) 24 (15)	1.00 0.83 (0.43-1.62)	0.592	1.00 0.80 (0.39-1.64)	0.539

^a^Adjusted for: T-stage, N-stage, R-classification

^b^Adjusted for: age, T-stage, N-stage, R-classification, differentiation grade

## Results

### Expression of IFITM1 in normal epithelium, intestinal metaplasia, primary tumors and lymph node metastases

**Fig. 1:**
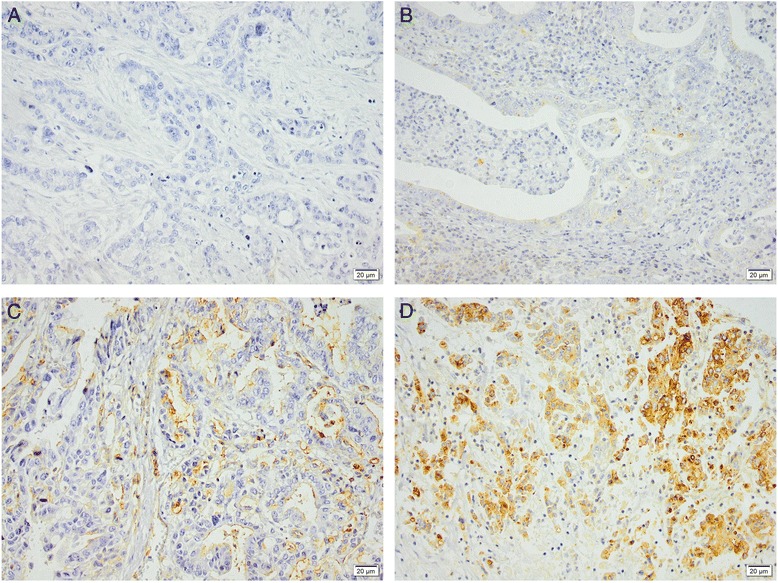
Sample immunohistochemical images of IFITM1 staining in gastroesophageal adenocarcinoma primary tumors with (**a**) negative, (**b**) weak, (**c**) moderate, and (**d**) strong staining of tumor cells. Magnification x 20

**Fig. 2:**
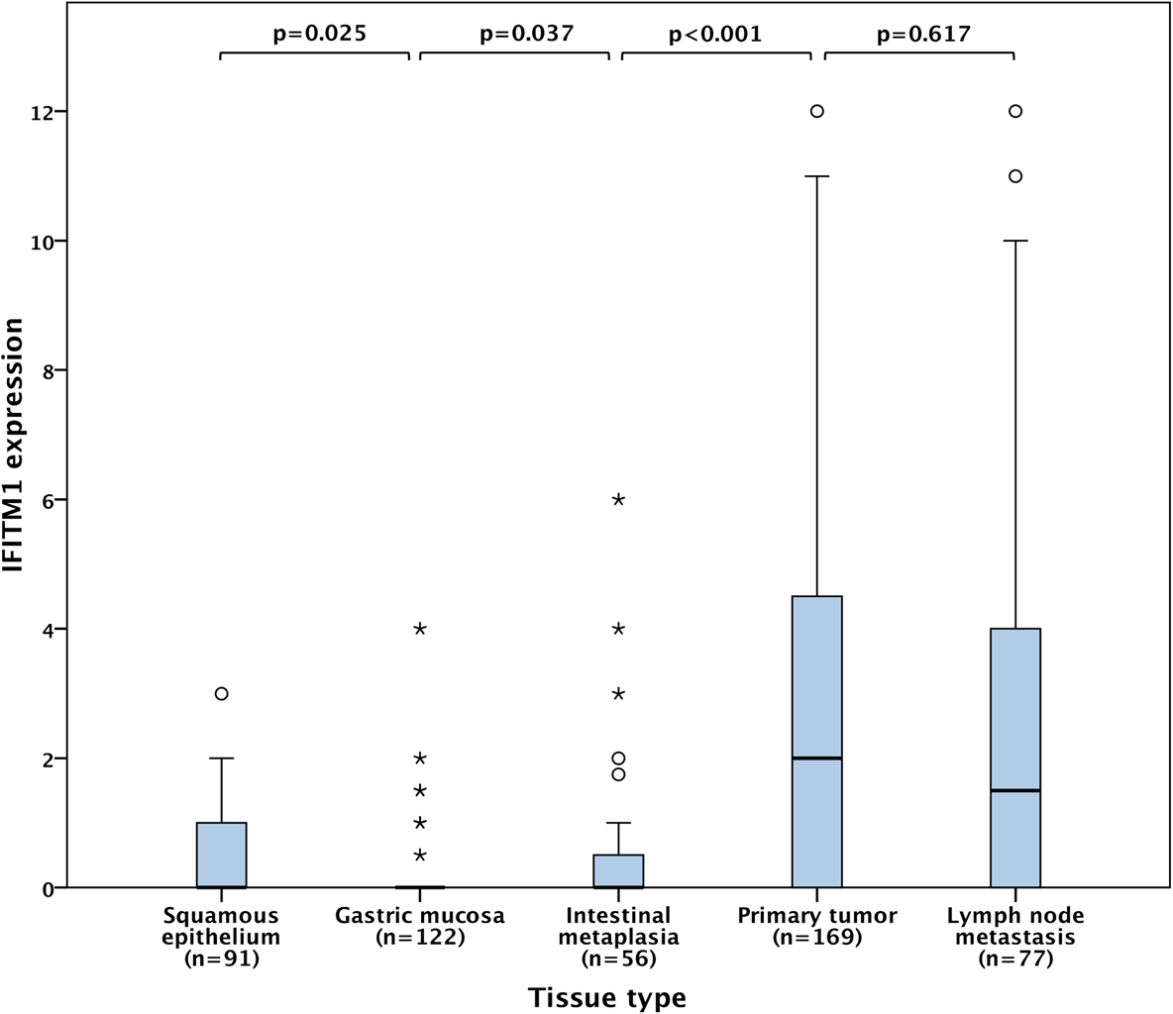
Box plots visualizing the distribution of immunohistochemical IFITM1 expression (fraction x intensity) in normal squamous epithelium, normal gastric mucosa, intestinal metaplasia (Barrett’s esophagus or gastric intestinal metaplasia), primary tumors and lymph node metastases in the entire cohort. The whiskers represent the largest values within 1.5 x interquartile range, the circles and asterisks represent outliers and extremes, respectively

Immunohistochemical expression of IFITM1 could be assessed in 91/96 (95 %) samples with esophageal squamous epithelium, 122/131 (93 %) samples with gastric mucosa, 56/73 (77 %) samples with intestinal metaplasia (gastric intestinal metaplasia or Barrett’s esophagus), 169/174 (97 %) samples with primary tumors, and 77/81 (95 %) samples with lymph node metastases. Sample images are shown in Fig. 1. The distribution of immunohistochemical expression of IFITM1 in the different tissue types is shown in Fig. 2. Expression of IFITM1 was significantly elevated in primary tumors and lymph node metastases compared to adjacent normal epithelium and intestinal metaplasia (Fig. 2). There were no significant differences of IFITM1 expression in primary tumors grouped by tumor location (Table 2).

### Associations of IFITM1 expression in primary tumors with clinicopathological data

Table 2 describes the expression of IFITM1 in primary tumors in relationship to clinicopathological data for the entire cohort and for the separate tumor locations. In the entire cohort, IFITM1 was significantly elevated in M0-disease, most notably in gastric cancer. There was a trend towards higher IFITM1 expression in primary tumors with a background of intestinal metaplasia. In the subset of gastric tumors there were significant associations of high IFITM1 expression with age and Lauren’s intestinal type, respectively.

### Impact of IFITM1 expression on survival

**Fig. 3:**
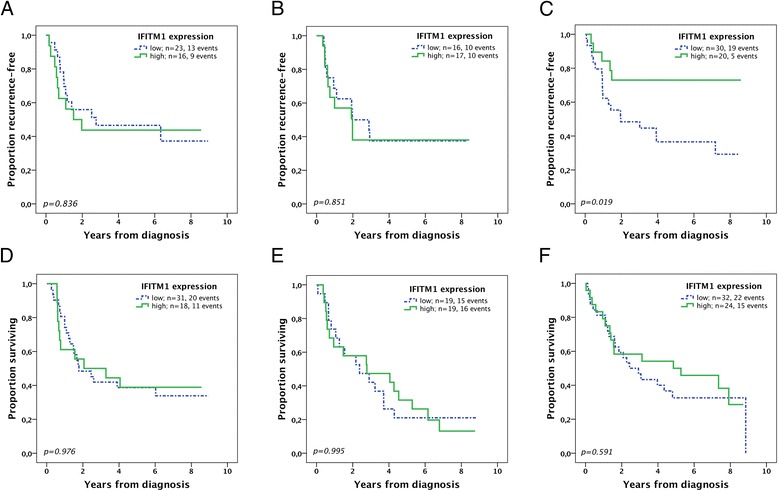
Kaplan-Meier plots of time to recurrence and overall survival according to IFITM1 expression in patients with M0-disease and no macroscopic residual tumor (R0-1). Time to recurrence in **a** esophageal cancer, **b** GE junction cancer, and **c** gastric cancer. Overall survival in **d** esophageal cancer, **e** GE junction cancer, and **f** gastric cancer

Survival analyses were performed on patients with M0-disease and no macroscopic residual tumor (R0-1). Using ROC-curves, both for the separate primary tumor locations and for the entire cohort and with regard to TTR as well as OS, an optimal cut-off at 3 (IFITM1 low < 3, IFITM1 high 3–12) was identified and subsequently used for both TTR and OS, irrespectively of tumor location. In esophageal adenocarcinoma, expression of IFITM1 had no impact on TTR and OS in the Kaplan-Meier-analyses (Fig. 3a, d), but in the adjusted Cox regression analyses (Table 3 and Additional file 1: Table S1)) high IFITM1 expression had a negative impact on both TTR (HR 3.05, 95 % CI 1.09-8.53, *p* = 0.034) and OS (HR 2.71, 95 % CI 1.11-6.67, *p* = 0.029). IFITM1 expression in GE junction tumors did not correlate with TTR or OS in neither Kaplan-Meier (Fig. 3b, e) nor Cox regression analyses (Table 3 and Additional file 1: Table S2). In gastric cancer, high IFITM1 expression was significantly associated with improved TTR in the Kaplan-Meier analyses (Fig. 3c, f) and Cox regression (Table 3 and Additional file 1: Table S3), both in the unadjusted analysis (HR 0.33, 95 % CI 0.12-0.88, *p* = 0.026) and in the adjusted analysis (HR 0.32, 95 % CI 0.12-0.87, *p* = 0.026) but there was no significant impact on OS.

Of note, considering the association of high IFITM1 with Lauren’s intestinal type in gastric cancer (Table 2), we also tested to replace IFITM1 with Lauren classification in the adjusted Cox regression model but the hazard ratio of Lauren classification for TTR was not significant (data not shown) and when we added Lauren classification to the model with IFITM1 the hazard ratio of IFITM1 on TTR remained significant (data not shown). Thus, we do not believe that IFITM1 is just a marker for Lauren’s intestinal type.

Of the 78 patients that developed recurrent disease during the follow-up period, 36 patients received palliative treatment with chemotherapy and/or radiotherapy. To what extent palliative therapy may have affected the outcome is unclear, but due to a considerable heterogeneity regarding treatment type, doses and duration as well as to avoid selection bias (patients offered active palliative treatment usually have better performance status and prognosis) we decided not to include palliative oncological treatment after recurrence as a variable in the survival analyses.

## Discussion

The current study showed a significantly increased expression of IFITM1 in gastroesophageal adenocarcinoma compared to adjacent normal epithelium. This finding is in accordance with other reports on IFITM1 in gastric and colorectal adenocarcinoma [23, 34, 35]. The association of high IFITM1 expression and M0-disease, particularly seen in gastric cancer, has to our knowledge not been described previously.

In gastric cancer with high expression of IFITM1, we have demonstrated consistent findings of a beneficial effect on TTR. However, we could not demonstrate any significant relationship between IFITM1 and OS in gastric cancer, and one possible explanation for this could be the older age in these patients. It has previously been suggested that IFITM1 may have an adverse impact on OS in gastric cancer [23] but, even though our data on OS were non-significant, the association of elevated IFITM1 with M0-disease and the favorable impact on TTR implies that high expression of IFITM1 could rather be a positive prognostic factor in gastric cancer. It may seem like a paradox that the overexpression of IFITM1 in gastric cancer, which in other malignancies has been shown to promote tumorigenesis, was associated with M0-disease and a favorable TTR. A possible explanation might be that gastric tumorigenesis associated with elevated IFITM1 confers a less malignant phenotype. Support for this is the observed association of high IFITM1 expression and the prognostically favorable Lauren’s intestinal type demonstrated both in this study and by others [23]. A similar contradiction has been described in glioma cells where knockdown of IFITM1 was demonstrated to inhibit proliferation, migration and invasion [17, 18], whereas reduced expression of IFITM1 correlated with shorter survival in a cohort of 30 glioma patients [17].

The proposed negative impact of IFITM1 on TTR and OS in esophageal adenocarcinoma has to be interpreted with caution since it was only demonstrated in the adjusted Cox regression analysis. However, if true, this would suggest the involvement of a different tumorigenic pathway than in gastric cancer. Esophageal and gastric cancers are indeed different malignancies, with diverging incidence trends and different risk factors. For instance, *Helicobacter pylori* infection is associated with gastric cancer [7] but may be a protective factor for esophageal cancer [5].

The exact function of IFITM1 in malignancy is poorly understood and its role might differ depending on tumor cell type and context. IFITM1 has been demonstrated to promote malignant progression in gastric cancer cells by increasing invasion and migration and by suppressing natural killer cell activity [23, 35]. It has been shown that IFITM1 expression is regulated by DNA methylation of its promoter region [23]. Furthermore, expression of a transcript of CDH1 (E-cadherin) intron 2 (CDH1a) has been shown to increase gastric cancer cell invasion and angiogenesis and this increase correlated with IFITM1 expression [36]. The downstream effectors of IFITM1 on tumorigenesis are largely unknown but one possible mechanism of promoting invasion could be the upregulation of matrix metalloproteinases [20].

An association between high IFITM1 expression and sensitivity to cisplatin has been described in esophageal squamous cell carcinoma [37] whereas in gastric cancer, overexpression of IFITM1 may confer resistance to cisplatin [38]. Thus, future studies, on patient cohorts treated with neoadjuvant or palliative chemotherapy, would be of interest to further assess the possible role of IFITM1 as a predictive biomarker for response to platinum-based chemotherapy.

A limitation of our study is the retrospective design. However, all available surgically resected tumors were included consecutively, which decreases the risk of selection bias, and all clinical and histopathological data have been thoroughly re-examined. Another possible limitation is the use of the TMA technique, but since duplicate cores were obtained from different donor blocks, the risk of sampling bias should be low. Moreover, analyzing the data grouped by tumor location reduces the sample size and number of events and thus limits the possibility to adjust for multiple possible confounders in the Cox regression analyses. Due to the exploratory nature of the study, our results should mainly be regarded as hypothesis-generating, providing a basis for further exploration of IFITM1 as a biomarker in gastroesophageal adenocarcinoma.

## Conclusion

In summary, we have shown that the immunohistochemical expression of IFITM1 was elevated in gastroesophageal adenocarcinoma and that it was associated with M0-disease. In gastric cancer, IFITM1 had a positive impact on TTR, whereas in esophageal cancer, data indicates an adverse impact on survival, suggesting that the role of IFITM1 may differ depending on the tumorigenic pathway. The mechanistic basis for this observation merits further study, and validatory studies on tumors from additional patient cohorts are warranted.

### Ethics approval

The study was approved by the regional ethics committee at Lund University (ref nr 445/07), whereby the committee waived the need for consent other than by the option to opt out.

## Additional file

Additional file 1: Table S1-S3. Hazard ratios for recurrence and death. Detailed Cox proportional-hazards regression for esophageal, GE junction and gastric adenocarcinoma. (DOCX 29 kb)

## Abbreviations

CI: confidence interval
GE: gastroesophageal
HR: hazard ratio
IFITM1: interferon-inducible transmembrane protein 1
OS: overall survival
ROC: receiver operating characteristic
TMA: tissue microarray
TTR: time to recurrence
