# Supplementary material for: Expression of IFITM1 as a prognostic biomarker in resected gastric and esophageal adenocarcinoma
Source: Biomark Res. 2016 May 14;4:10. doi: 10.1186/s40364-016-0064-5 (PMC4867989; doi:10.1186/s40364-016-0064-5)
Supplement: Additional file 1: Table S1-S3. — Hazard ratios for recurrence and death. Detailed Cox proportional-hazards regression for esophageal, GE junction and gastric adenocarcinoma. (DOCX 29 kb) [file 40364_2016_64_MOESM1_ESM.docx]

| **Table S1. Hazard ratios for recurrence and death ESOPHAGUS** | | | | | | | | | | |
| --- | --- | --- | --- | --- | --- | --- | --- | --- | --- | --- |
|  | **Time to recurrence (M0 R0-1)** | | | | | **Overall survival (M0 R0-1)** | | | | |
|  |  | **Unadjusted** | | **Adjusted** | |  | **Unadjusted** | | **Adjusted** | |
|  | n (events) | HR (95% CI) | p-value | HR (95% CI) | p-value | n (events) | HR (95% CI) | p-value | HR (95% CI) | p-value |
| **Age**  continuous | 41 (22) | 1.03 (0.99-1.08) | 0.106 |  |  | 51 (31) | 1.05 (1.01-1.09) | 0.009 | 1.05 (1.01-1.09) | 0.011 |
| **Sex**  Female  Male | 3 (2)  38 (20) | 1.00  0.63 (0.15-2.69) | 0.528 |  |  | 5 (4)  46 (27) | 1.00  0.53 (0.18-1.53) | 0.241 |  |  |
| **T-stage**  T1-2  T3-4 | 14 (2)  26 (20) | 1.00  8.14 (1.90-34.96) | 0.005 | 1.00  1.03 (0.14-7.45) | 0.977 | 17 (7)  33 (24) | 1.00  2.76 (1.18-6.43) | 0.019 | 1.00  0.98 (0.26-3.62) | 0.972 |
| **N-stage**  N0  N1-3 | 14 (2)  27 (20) | 1.00  8.13 (1.89-34.90) | 0.005 | 1.00  8.68 (1.25-60.19) | 0.029 | 14 (4)  37 (27) | 1.00  4.14 (1.44-11.88) | 0.008 | 1.00  4.77 (1.28-17.73) | 0.020 |
| **R-classification**  R0  R1 | 27 (9)  14 (13) | 1.00  5.85 (2.43-14.10) | <0.001 | 1.00  4.12 (1.44-11.79) | 0.008 | 33 (14)  18 (17) | 1.00  3.99 (1.91-8.35) | <0.001 | 1.00  3.13 (1.12-8.77) | 0.030 |
| **Differentiation grade**  High/Moderate  Low | 22 (9)  19 (13) | 1.00  2.37 (1.01-5.56) | 0.048 |  |  | 26 (11)  25 (20) | 1.00  2.45 (1.17-5.14) | 0.018 | 1.00  1.23 (0.50-3.01) | 0.657 |
| **Lauren classification**  Intestinal  Diffuse/Mixed | 36 (17)  5 (5) | 1.00  3.97 (1.43-11.01) | 0.008 |  |  | 45 (25)  6 (6) | 1.00  2.11 (0.85-5.24) | 0.109 |  |  |
| **Intestinal metaplasia background**  No  Yes | 25 (15)  16 (7) | 1.00  0.66 (0.27-1.61) | 0.357 |  |  | 31 (20)  20 (11) | 1.00  0.70 (0.33-1.46) | 0.342 |  |  |
| **Adjuvant treatment**  No  Yes | 38 (20)  3 (2) | 1.00  1.41 (0.33-6.04) | 0.647 |  |  | 47 (28)  4 (3) | 1.00  1.67 (0.51-5.52) | 0.399 |  |  |
| **IFITM1**  Low (<3)  High (3-12) | 23 (13)  16 (9) | 1.00  1.09 (0.47-2.56) | 0.836 | 1.00  3.05 (1.09-8.53) | 0.034 | 31 (20)  18 (11) | 1.00  0.99 (0.47-2.07) | 0.976 | 1.00  2.71 (1.11-6.67) | 0.029 |

| **Table S2. Hazard ratios for recurrence and death GE JUNCTION** | | | | | | | | | | |
| --- | --- | --- | --- | --- | --- | --- | --- | --- | --- | --- |
|  | **Time to recurrence (M0 R0-1)** | | | | | **Overall survival (M0 R0-1)** | | | | |
|  |  | **Unadjusted** | | **Adjusted** | |  | **Unadjusted** | | **Adjusted** | |
|  | n (events) | HR (95% CI) | p-value | HR (95% CI) | p-value | n (events) | HR (95% CI) | p-value | HR (95% CI) | p-value |
| **Age**  continuous | 34 (20) | 0.97 (0.93-1.01) | 0.124 |  |  | 39 (31) | 1.01 (0.98-1.04) | 0.568 | 1.04 (0.99-1.08) | 0.094 |
| **Sex**  Female  Male | 6 (2)  28 (18) | 1.00  1.81 (0.42-7.80) | 0.428 |  |  | 9 (8)  30 (23) | 1.00  0.52 (0.23-1.17) | 0.116 |  |  |
| **T-stage**  T1-2  T3-4 | 7 (2)  26 (17) | 1.00  2.79 (0.64-12.14) | 0.171 | 1.00  2.34 (0.52-10.45) | 0.266 | 7 (4)  31 (26) | 1.00  1.95 (0.68-5.59) | 0.217 | 1.00  1.70 (0.55-5.23) | 0.355 |
| **N-stage**  N0  N1-3 | 10 (2)  24 (18) | 1.00  5.86 (1.35-25.41) | 0.018 | 1.00  7.68 (1.64-36.12) | 0.010 | 11 (8)  28 (23) | 1.00  1.61 (0.72-3.62) | 0.250 | 1.00  2.66 (0.95-7.46) | 0.063 |
| **R-classification**  R0  R1 | 25 (13)  9 (7) | 1.00  2.05 (0.81-5.20) | 0.131 | 1.00  2.79 (0.98-7.95) | 0.055 | 26 (19)  13 (12) | 1.00  2.58 (1.22-5.47) | 0.013 | 1.00  2.45(1.03-5.78) | 0.042 |
| **Differentiation grade**  High/Moderate  Low | 13 (5)  21 (15) | 1.00  2.43 (0.87-6.73) | 0.089 |  |  | 13 (8)  26 (23) | 1.00  2.06 (0.92-4.63) | 0.081 | 1.00  1.49 (0.59-3.78) | 0.402 |
| **Lauren classification**  Intestinal  Diffuse/Mixed | 24 (14)  10 (6) | 1.00  0.92 (0.35-2.40) | 0.864 |  |  | 27 (21)  12 (10) | 1.00  1.08 (0.51-2.30) | 0.843 |  |  |
| **Intestinal metaplasia background**  No  Yes | 24 (15)  10 (5) | 1.00  0.69 (0.25-1.91) | 0.478 |  |  | 29 (23)  10 (8) | 1.00  0.72 (0.32-1.62) | 0.432 |  |  |
| **Adjuvant treatment**  No  Yes | 31 (18)  3 (2) | 1.00  1.10 (0.25-4.77) | 0.90 |  |  | 36 (29)  3 (2) | 1.00  0.57 (0.14-2.40) | 0.445 |  |  |
| **IFITM1**  Low (<3)  High (3-12) | 16 (10)  17 (10) | 1.00  1.09 (0.45-2.62) | 0.852 | 1.00  1.50 (0.59-3.82) | 0.400 | 19 (15)  19 (16) | 1.00  1.00 (0.49-2.03) | 0.995 | 1.00  0.97 (0.44-2.15) | 0.937 |

| **Table S3. Hazard ratios for recurrence and death STOMACH** | | | | | | | | | | |
| --- | --- | --- | --- | --- | --- | --- | --- | --- | --- | --- |
|  | **Time to recurrence (M0 R0-1)** | | | | | **Overall survival (M0 R0-1)** | | | | |
|  |  | **Unadjusted** | | **Adjusted** | |  | **Unadjusted** | | **Adjusted** | |
|  | n (events) | HR (95% CI) | p-value | HR (95% CI) | p-value | n (events) | HR (95% CI) | p-value | HR (95% CI) | p-value |
| **Age**  continuous | 50 (24) | 1.00 (0.97-1.03) | 0.906 |  |  | 57 (38) | 1.05 (1.02-1.09) | 0.001 | 1.07 (1.03-1.11) | <0.001 |
| **Sex**  Female  Male | 15 (6)  35 (18) | 1.00  1.41 (0.56-3.55) | 0.470 |  |  | 17 (10)  40 (28) | 1.00  1.26 (0.61-2.62) | 0.530 |  |  |
| **T-stage**  T1-2  T3-4 | 22 (7)  27 (16) | 1.00  2.50 (1.01-6.16) | 0.047 | 1.00  1.43 (0.53-3.89) | 0.481 | 25 (15)  31 (22) | 1.00  1.69 (0.86-3.32) | 0.132 | 1.00  0.97 (0.44-2.14) | 0.933 |
| **N-stage**  N0  N1-3 | 22 (3)  28 (21) | 1.00  10.74 (3.15-36.62) | <0.001 | 1.00  10.39 (2.96-36.49) | <0.001 | 26 (13)  31 (25) | 1.00  2.78 (1.39-5.56) | 0.004 | 1.00  3.33 (1.50-7.37) | 0.003 |
| **R-classification**  R0  R1 | 46 (20)  4 (4) | 1.00  3.07 (1.02-9.31) | 0.047 | 1.00  2.52 (0.69-9.23) | 0.162 | 52 (33)  5 (5) | 1.00  3.36 (1.24-9.14) | 0.018 | 1.00  3.15 (0.97-10.27) | 0.241  0.057 |
| **Differentiation grade**  High/Moderate  Low | 14 (3)  36 (21) | 1.00  2.99 (0.89-10.04) | 0.077 |  |  | 15 (11)  42 (27) | 1.00  0.88 (0.43-1.78) | 0.720 | 1.00  1.40 (0.62-3.17) | 0.425 |
| **Lauren classification**  Intestinal  Diffuse/ Mixed | 27 (10)  23 (14) | 1.00  1.96 (0.87-4.42) | 0.104 |  |  | 31 (21)  26 (17) | 1.00  0.97 (0.51-1.87) | 0.937 |  |  |
| **Intestinal metaplasia background**  No  Yes | 23 (11)  27 (13) | 1.00  1.04 (0.47-2.33) | 0.920 |  |  | 24 (17)  33 (21) | 1.00  1.02 (0.53-1.95) | 0.958 |  |  |
| **Adjuvant treatment**  No  Yes | 45 (19)  5 (5) | 1.00  3.13 (1.15-8.51 | 0.025 |  |  | 52 (33)  5 (5) | 1.00  1.73 (0.61-4.92) | 0.308 |  |  |
| **IFITM1**  Low (<3)  High (3-12) | 30 (19)  20 (5) | 1.00  0.33 (0.12-0.88) | 0.026 | 1.00  0.32 (0.12-0.87) | 0.026 | 32 (22)  24 (15) | 1.00  0.83 (0.43-1.62) | 0.592 | 1.00  0.80 (0.39-1.64) | 0.539 |
